# Application of an Incremental Constitutive Model for the FE Analysis of Material Dynamic Restoration in the Rotary Tube Piercing Process

**DOI:** 10.3390/ma13194289

**Published:** 2020-09-25

**Authors:** Alberto Murillo-Marrodán, Eduardo García, Jon Barco, Fernando Cortés

**Affiliations:** 1 Department of Mechanics, Design and Industrial Management, University of Deusto, Avda Universidades 24, 48007 Bilbao, Spain; e.garcia@deusto.es (E.G.); fernando.cortes@deusto.es (F.C.); 2 R&D Department, Tubos Reunidos Industrial, Barrio Sagarribai 2, 01470 Amurrio, Spain; Jbarco@tubosreunidos.com

**Keywords:** incremental model, constitutive model, metal forming, rotary tube piercing, FE analysis, tube eccentricity

## Abstract

In the numerical simulation of hot forming processes, the correct description of material flow stress is very important for the accuracy of the results. For complex manufacturing processes, such as the rotary tube piercing (RTP), constitutive laws based on both power and exponential mathematical expressions are commonly used due to its inherent simplicity, despite the limitations that this approach involves, namely, the use of accumulated strain as a state parameter. In this paper, a constitutive model of the P91 steel derived from the evolution of dislocation density with strain, which takes into account the mechanisms of dynamic recovery (DRV) and dynamic recrystallization (DRX), is proposed for the finite element (FE) analysis of the RTP process. The material model is developed in an incremental manner to allow its implementation in the FE code FORGE^®^. The success of this implementation is confirmed by the good correlation between results of the simulation and experimental measurements of the manufactured tube (elongation, twist angle, mean wall thickness and eccentricity). In addition, this incremental model allows addressing how the restoring mechanisms of DRV and DRV occur during the RTP process. The analysis puts into evidence that DRV and DRX prevail over each other cyclically, following an alternating sequence during the material processing, due mainly to the effect of the strain rate on the material.

## 1. Introduction

The correct numerical modeling of the flow behaviour of metals in hot deformation simulation is fundamental for the analysis of metal forming processes, because of its importance on metal flow pattern as well as the kinetics of metallurgical transformation (for example, static, dynamic, and meta-dynamic recrystallization behaviour) [1]. Generally, constitutive relations are used to describe the plastic flow properties of the metals and alloys in a form that can be used in computer code.

In the particular case of the Rotary Tube Piercing (RTP) of seamless tubes, this complex process is conducted under high temperature with a range of 1200–1300 °C, strain could vary in the range of 500 to 1000% and the strain rate in the span of 40 to 50 s^−1^ [2]. Therefore, for its numerical analysis, the reliability of the results depends on the constitutive relation used for the description of the material behaviour under these severe deformation conditions.

In many of the reviewed scientific contributions, the constitutive model is not described. On the other hand, in some of them, it is only specified if a rigid-plastic, rigid-viscoplastic, elasto-plastic or elasto-viscoplastic approach is considered [3,4,5,6,7,8,9]. In addition, other authors claim that they have used the material constitutive model from the FE software database without giving details about the specific formulation used, or only state that the flow stress was dependent on the temperature and the strain rate [10,11,12,13,14,15,16,17,18,19,20]. In those studies in which information about the constitutive material formulation is provided, the constitutive formulations are commonly dependent on the strain, strain rate and temperature of the material through the use of both power and exponential based mathematical expressions. Among them the following stand out:

- Hansel–Spittel constitutive model: this model is given by [21].
  (1) $$\sigma \left(\bar{\varepsilon },\dot{\bar{\varepsilon }},T\right)=A\exp\left(m_{1}T\right){\bar{\varepsilon }}^{m_{2}}{\dot{\bar{\varepsilon }}}^{m_{3}}\exp\left(\frac{m_{4}}{\bar{\varepsilon }}\right),$$
  where *A* and the $m_{i}$ are material parameters determined from experimental tests, $\bar{\varepsilon }$ is the equivalent strain, $\dot{\bar{\varepsilon }}$ is the equivalent strain rate and *T* is the temperature. It is a widespread formulation, which is one of the available models in the software FORGE^®^ (NxT 2.1, Transvalor, Mougins, France). This constitutive formulation is valid for both high and low temperature and strain rate [22].
  Regarding the RTP process, the Hansel–Spittel law has been frequently used for the constitutive modelling of the billet material behaviour. Ghiotti and Fanini [23,24,25] modelled the flow stress of a DIN St52 steel in a skew rolling process with this formulation. Pschera et al. [26] used this constitutive formulation for modelling the 10CrMo9-10 steel rheological behaviour during the RTP and the posterior analysis of material damage. Pater et al. [27] used also this model to compare the RTP process in two and three-roll mills. Murillo-Marrodán et al. [28,29] used this formulation for the analysis of the RTP process, reproducing accurately the tube deformation.
  There is also an extended version of Hansel–Spittel. This extended constitutive model is similar to the previously described one, but incorporates more terms in which temperature, strain rate and strain are coupled, yielding
  (2) $$\sigma \left(\bar{\varepsilon },\dot{\bar{\varepsilon }},T\right)=A\exp\left(m_{1}T\right){\bar{\varepsilon }}^{m_{2}}{\dot{\bar{\varepsilon }}}^{m_{3}}\exp\left(\frac{m_{4}}{\bar{\varepsilon }}\right){\left(1+\bar{\varepsilon }\right)}^{m_{5}T}\exp\left(m_{7}\bar{\varepsilon }\right){\dot{\bar{\varepsilon }}}^{m_{8}T}T^{m_{9}},$$
  where $m_{5}$ defines the coupling of strain and temperature through a potential term, $m_{7}$ is the exponential dependence of stress on strain, $m_{8}$ corresponds to the coupling between strain rate and temperature through a power relation and $m_{9}$ is the power dependence of stress on temperature. The application of this extended model in the RTP process was used to analyze the material deformation [30] for billets made of St52, 10CrMo9-10 and X10CrMoVNb9-1 (P91) steels. However, this model is more complex and requires additional time for convergence and additional resources to determine a higher number of material parameters.

- Modified versions of the Hansel–Spittel constitutive model: Chastel et al. [31] used a simplification of the model, given by
  (3) $$\sigma \left(\bar{\varepsilon },\dot{\bar{\varepsilon }},T\right)=A\exp\left(m_{1}T\right){\bar{\varepsilon }}^{m_{2}}{\dot{\bar{\varepsilon }}}^{m_{3}},$$
  with similar results to those of Hansel–Spittel in the analyzed process conditions.
  Pater et al. [32] used a modified expression of the Hansel–Spittel law, for 100Cr6 steel, in which flow stress dependence on strain was altered. The flow stress was calculated according to
  (4) $$\sigma \left(\bar{\varepsilon },\dot{\bar{\varepsilon }},T\right)=A_{0}+A\exp\left(m_{1}T\right){\bar{\varepsilon }}^{m_{2}}{\dot{\bar{\varepsilon }}}^{m_{3}}\exp\left(m_{7}\bar{\varepsilon }\right),$$
  where $A_{0}$ is a material constant that is related to the yield stress of the material. It was compared to the experiments performed, showing good precision.
  Lu et al. [33,34,35] simulated the flow stress of the 33Mn2V steel in the RTP process with an alternative constitutive law adopted from [36], yielding
  (5) $$\sigma \left(\bar{\varepsilon },\dot{\bar{\varepsilon }},T\right)=A\exp\left(m_{1}T\right){\bar{\varepsilon }}^{m_{2}}{\dot{\bar{\varepsilon }}}^{\left(m_{3}+m_{8}T\right)}.$$
  The results of force were assessed using this material model showing good agreement with the experimental data.

- Arrhenius-type constitutive model: the hyperbolic sine law in the Arrhenius-type equation gives a good approximation of the Zener–Hollomon parameter *Z* and flow stress, and can be used for a wide range of deformation conditions [37]. Therefore, for all stress levels, the hyperbolic sine law can be represented as
  (6) $$\dot{\varepsilon }=B\sinh^{n}\left(\alpha \sigma \right)\exp\left(\frac{-Q}{RT}\right),$$
  where the flow stress given by the hyperbolic sine law can be calculated as a function of *Z*, yielding
  (7) $$\sigma =\frac{1}{\alpha }\ln\left[{\left(\frac{Z}{B}\right)}^{\frac{1}{n}}+{\left[{\left(\frac{Z}{B}\right)}^{\frac{2}{n}}+1\right]}^{\frac{1}{2}}\right],$$
  and *B*, α and *n* are the material parameters dependent on the actual strain. In this constitutive approach, the flow softening of the material is determined by the value of the Zener–Hollomon parameter, *Z* [38]. This parameter incorporates the effects of the temperature and strain rate on the deformation behaviour according to
  (8) $$Z=\dot{\varepsilon }\exp\left(\frac{Q}{RT}\right),$$
  where R is the universal gas constant and *Q* the apparent activation energy of the material. The lower the value of *Z*, the higher the softening of the material. This constitutive model was used by Ding et al. [39] for modelling the AZ31 magnesium alloy behaviour in a rotary piercing operation. Zhao et al. [40] proposed a modification of Equation (6) for studying the flow stress and dynamic softening of a 55SiMnMo steel, yielding
  (9) $$\dot{\varepsilon }=B\exp\left(\alpha \sigma \right)\exp\left(\frac{-Q}{RT}\right).$$

In short, one important drawback of these models is the dependence of the flow stress on the actual strain, which has been shown not to be a valid state variable [41]. Nevertheless, in previous studies of the RTP process [28,29,42,43], Hansel–Spittel has been used, providing accurate results compared to experimental data, but this model does not allow for analyzing the softening processes that take place during plastic deformation.

By contrast, there are constitutive formulations that consider mechanisms such as the thermally activated dislocation movement for the description of the constitutive relations between flow stress and plastic strain. These models are based on valid state parameters and allow the analysis of the material dynamic restoration mechanisms. In the literature, these constitutive formulations are available for different materials such as stainless steels [44,45], nickel-based superalloys [46], titanium alloys [47,48] or aluminum alloys [49,50]. However, these models have only been succesfully applied to the FE simulation of cutting operations [51,52]. Therefore, a lack of such constitutive models ready to be used in the simulation of the rotary tube piercing process was identified. For this reason, in this paper, a constitutive model based on the Estrin–Mecking (EM) equation [53] has been developed as an incremental formulation, which has been implemented in the FE software FORGE^®^ to simulate the RTP process. This model is able to accurately predict the flow stress, work-hardening and work-softening rate of the P91 steel both under constant and transient deformation conditions because the flow stress is not dependent on the strain, but on the strain path during the plastic deformation. The deformation of the tube, including the wall thickness eccentricity that is one of the most important geometrical parameters of the conformed tube, is analyzed as a result of the simulation. Finally, the incremental constitutive model is used to assess the proposed deformation mechanisms involved in the RTP process, namely, dynamic recovery (DRV) and dynamic recrystallization (DRX).

## 2. Theoretical Background of the Constitutive Model

The constitutive model of the P91 steel was developed from an experimental characterization of the material. In this model, the description of the material dynamic recovery (DRV) and the work-hardening (WH) is given by an EM equation. The basis of this model is the dislocation density evolution with respect to the plastic strain applied to the material ε, derived by Estrin and Mecking [53] according to

(10) $$\frac{d\rho }{d\varepsilon }=h-r\rho ,$$

where *h* is the athermal dislocation storage rate, ρ is the material dislocation density and *r* the dynamic recovery rate. The flow stress σ is related to the dislocation density of the material resulting in

(11) $$\sigma =\alpha \mu_{s}b\rho^{\frac{1}{2}},$$

where *b* represents the Burgers vector, $\mu_{s}$ the shear modulus and α≈1 [54]. From Equation (11), Jonas et al. [55] defined a relation of proportionality between the flow stress σ and the dislocation density ρ. On this basis, the model has been proposed as an step-by-step incremental constitutive formulation, suitable for its implementation in finite element codes [56]. The flow stress during the work-hardening and DRV transient, $\sigma_{\mathrm{DRV}}$, is given by

(12) $$\sigma_{\mathrm{DRV}}^{\left(j\right)}={\left[{\left(\sigma_{S}^{\left(j\right)}\right)}^{2}-\left({\left(\sigma_{S}^{\left(j\right)}\right)}^{2}-{\left(\sigma_{\mathrm{DRV}}^{\left(j-1\right)}\right)}^{2}\right)\exp\left(\frac{-2\theta_{0}\Delta \varepsilon^{\left(j\right)}}{\sigma_{S}^{\left(j\right)}}\right)\right]}^{\frac{1}{2}},$$

with

(13) $$\sigma_{\mathrm{DRV}}^{\left(1\right)}=\sigma_{Y}^{\left(1\right)},$$

where *j* is the step number (j≥2), $\sigma_{S}$ the saturation stress, $\sigma_{Y}$ the yield stress and $\theta_{0}$ the initial work-hardening rate, which is determined according to

(14) $$\frac{\theta_{0}}{\mu_{s}\left(T\right)}=k_{\theta }Z^{m_{\theta }},$$

where $k_{\theta }$ and $m_{\theta }$ are material constants. The temperature-dependent modulus $\mu_{s}\left(T\right)$ is computed according to Kocks [57] as

(15) $$\mu_{s}\left(T\right)=88884.6-37.3T,$$

where *T* is the temperature in Kelvin.

According to this formulation, both strain rate and temperature are computed after each step and both the WH and DRV are updated in each step from their previous value. The Sellars-Tegart-Garofalo (STG) model [36], is used to calculate the steady-state stress $\sigma_{\mathrm{SS}}$, saturation stress $\sigma_{S}$ and yield stress $\sigma_{Y}$ at each step, resulting in

(16) $$\sigma_{Y}\left(\dot{\varepsilon },T\right)=\sigma_{a}+\delta_{Y}\sinh^{-1}{\left(\frac{Z}{B_{Y}}\right)}^{\frac{1}{m_{Y}}},$$

(17) $$\sigma_{S}\left(\dot{\varepsilon },T\right)=\delta_{S}\sinh^{-1}{\left(\frac{Z}{B_{S}}\right)}^{\frac{1}{m_{S}}},$$

(18) $$\sigma_{\mathrm{SS}}\left(\dot{\varepsilon },T\right)=\delta_{\mathrm{SS}}\sinh^{-1}{\left(\frac{Z}{B_{\mathrm{SS}}}\right)}^{\frac{1}{m_{\mathrm{SS}}}}.$$

As previously mentioned in Section 1, *Z* is the Zener–Hollomon parameter, while $\sigma_{a}$ corresponds to the athermal stress and $\delta_{Y}$, $B_{Y}$, $m_{Y}$, $\delta_{S}$, $B_{S}$, $m_{S}$, $\delta_{\mathrm{SS}}$, $B_{\mathrm{SS}}$, $m_{\mathrm{SS}}$ are material constants. In order to compute the material softening due to DRX, a condition for the onset of dynamic recrystallization is required ($\sigma_{c}\approx \sigma_{\mathrm{SS}}$). In addition, the critical Zener–Hollomon parameter for the P91 steel is determined $Z_{c}=1.1\times 10^{15}s^{-1}$. This parameter defines if the material undergoes DRV $\left(Z\geq Z_{c}\right)$ or if DRX is the prevailing restoration mechanism $\left(Z<Z_{c}\right)$ depending on the deformation conditions. Therefore, both are necessary conditions for the onset of DRX. The time required for attaining a recrystallized fraction of a 50 % is calculated according to

(19) $$t_{50}=DZ^{-q}\exp\left(\frac{Q_{\mathrm{DRX}}}{RT}\right),$$

where *D* is a material parameter, which is dependent on the initial austenitic grain size of the billet of 120 μm, *q* is a material constant and $Q_{\mathrm{DRX}}$ is the apparent activation energy for hot deformation of 431.7 kJ · mol^−1^. Then, the recrystallized volume fraction is incrementally determined using

(20) $$X_{v}=1-\exp\left(-0.693{\left(\sum_{i=N_{0}}^{j}\frac{\Delta t^{\left(j\right)}}{t_{50}^{\left(j\right)}}\right)}^{n_{\mathrm{Av}}}\right),$$

where

(21) $$\Delta t^{\left(j\right)}=\frac{\Delta \varepsilon^{\left(j\right)}}{{\left(\dot{\varepsilon }\right)}^{\left(j\right)}}.$$

$N_{0}$ represents the increment at which DRX is initiated, and the material parameter $n_{\mathrm{Av}}=2$. Equation (12) is valid when only work hardening and dynamic recovery restoration mechanisms are active $\left(\sigma_{\mathrm{DRV}}<\sigma_{\mathrm{SS}}\right)$. Nevertheless, when the stress overcomes the critical value for the onset of DRX $\left(\sigma_{\mathrm{DRV}}\geq \sigma_{\mathrm{SS}}\right)$, the flow stress is determined according to

(22) $$\sigma^{\left(j\right)}=\sigma_{\mathrm{DRV}}^{\left(j\right)}-\left(\sigma_{S}^{\left(j\right)}-\sigma_{\mathrm{SS}}^{\left(j\right)}\right)X_{v}^{\left(j\right)}.$$

The methodology followed for the determination of all the material parameters involved in the constitutive model is available in [56]. The values of these material parameters are provided in Table 1, presenting an average absolute relative error (AARE) of approximately 4% with respect to the experimental data.

The incremental model was implemented in FORGE^®^, which extends it to a 3D formulation of stress and finite strain [58]. This implementation was validated by the 3D numerical simulation of compression tests [56].

## 3. FE Model of the RTP Process

**Figure 1:**
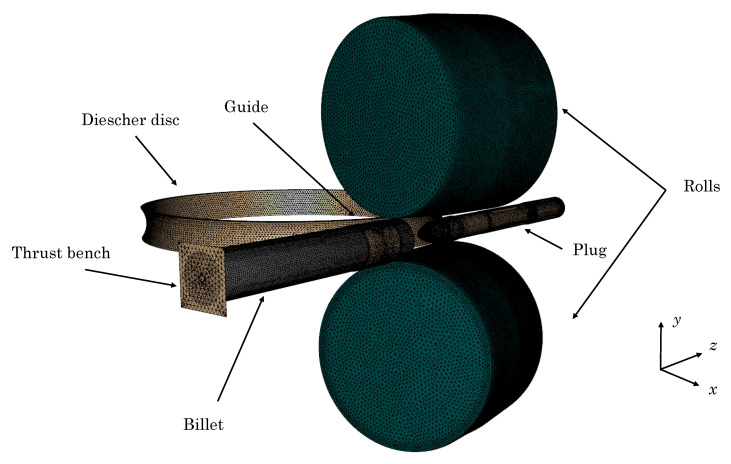
Meshing and layout of the rotary tube piercing mill (one of the lateral Diescher discs is not displayed).

The FE model of the RTP process is introduced in this section. This model is based on a previous study of the authors in which the Hansel–Spittel model was used [29]. The RTP process is performed in a piercing mill. This mill is made up of two rolls with a tapered shape, two lateral Diescher discs, the plug and a guide. The mesh and the position of these elements in the piercing mill is presented in Figure 1.

The billet is a round cylinder of 202 mm diameter and 1210 mm length. In order to reproduce the Mannesmann effect [23], a hollow cavity of 20 mm was defined in the billet axis. The guide, which is positioned coaxially to both the billet and the plug, conducts the billet during the process. At the beginning of the operation, the thrust bench contacts the billet and makes it advance in the piercing direction *z*. The billet is displaced in the axial direction *z* until contacting the rolls. The tapered rolls subject the billet to a combined movement of axial displacement and rotation because of their disposition. They rotate in a similar direction and present a feed angle β that misaligns their rotation axis leading to the aforementioned movement in the billet. The plug is responsible for the internal hollow space of the tube, while the lateral Dieschers contain the lateral expansion of the material. In addition, they rotate in opposite directions, which enhances the advance of the material in the *z* direction and avoids the ovalization of the tube. The geometry and kinematics of the elements of the RTP mill are listed in Table 2.

The guide, lateral discs, thrust bench and rolls have been modelled as rigid dies, while the plug is considered elastic with modulus E=190 GPa.

The billet was meshed with a total number of 300,000 3D tetrahedral P1+ linear elements, while for those elements modelled as rigid, 2D triangular elements were used.

The temperature distribution in the billet cross-section is defined, taking into account the transfer time between the furnace and the RTP mill. The ambient temperature was set to 50 °C. The temperature of the dies was 50 °C and the temperature of the plug was 500 °C. The thermal conductivity and specific heat of the billet are set from [59]. The thermal expansion coefficient was 1 × 10^−5^ °C. Regarding friction conditions, a constant shear friction model was used for the contact between the billet and the thrust bench, guide, Dieschers and plug. The friction conditions were defined based on previous research [29], yielding a friction coefficient for both the thrust bench and guide of m=0 (sliding conditions), while m=0.1 in the Dieschers and m=0.05 in the plug. The Norton friction model was used for modelling the rolls-billet contact with a friction coefficient α=0.22. The emissivity of the billet surface material was 0.5, the contact conductance was 10,000 W/m^2^K and natural convection was assumed, with a heat transfer coefficient of 10 W/m^2^K.

## 4. Results and Discussion

In this section, the numerical results relative to the tube deformation are compared to experimental measures taken from industrial RTP tubes [29] and the results provided by means of the Hansel–Spittel model [29]. Then, the material deformation conditions are analyzed in order to discern which dynamic restoration mechanism prevails at each region of the tube during the RTP process.

The analyzed results concerning the final tube geometry are the elongation $\epsilon_{z}$, the longitudinal torsion dψ/dz, the average wall thickness $t_{tubeavg}$ and the eccentricity $e_{c}$. The elongation is the relative increment of the tube length; the longitudinal torsion is related with the shear stress and it was measured as an angle per unit of length; the mean wall thickness was measured as an average value in 10 different sections of the tube, by means of 8 measures of thickness in each section. The wall thickness eccentricity, which is an imperfection inherent to the RTP process due to the loss of concentricity of the outer and inner walls of the tube, is determined according to

(23) $$e_{c}\left(\%\right)=\frac{t_{max}-t_{min}}{t_{avg}}\times 100,$$

where $t_{max}$ and $t_{min}$ are the maximum and minimum thickness of the tube in each cross-section, respectively, and $t_{avg}$ is the mean value of wall thickness at each cross-section.

The results provided by the simulation with the incremental model are compared to the experimental measures and the results of the previous study using Hansel–Spittel in Table 3.

**Figure 2:**
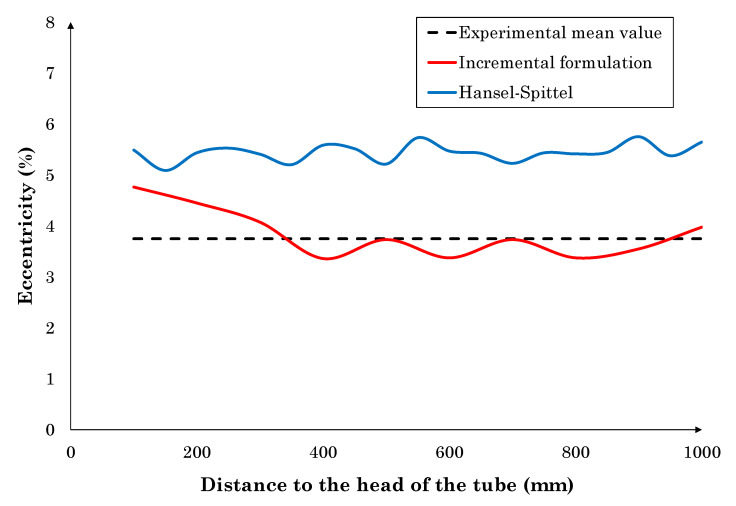
Numerical and mean experimental results of wall thickness eccentricity.

From the results, it can be pointed out that the maximum difference of the incremental dislocation density-based formulation corresponds to the numerical simulation of the tube longitudinal torsion, but it is considered acceptable given that the experimental measures are taken directly from the industrial process. Otherwise, the Hansel–Spittel formulation shows the maximum difference in the wall thickness eccentricity. This is one of the most important process flaws to take into account, given that it is subsequently transferred downstream through the manufacturing stages to the final product. The wall thickness eccentricity is associated to the combined effect of the uneven heating of the billet and the deformation of the plug. The uneven temperature distribution in the billet cross-section yields to softer material in the region with higher temperature. Therefore, the plug tends to be deformed towards this softer region because forces are not balanced. In additon, during the rotation of the billet, the plug follows this soft region, which is the cause of the eccentricity [29]. Therefore, the incremental model that takes into account the work hardening and also the dynamic softening of the material would provide results closer to the experimental value. The numerical wall thickness eccentricity evolution of the model using the incremental formulation and Hansel–Spittel are compared to the mean experimental value in Figure 2.

This figure is aimed at showing that wall thickness eccentricity results are uniform along the measured length of the tube. Besides, the incremental formulation results present the lowest error. This can be associated with the correct reproduction of the material work hardening and work softening, in contrast to Hansel–Spittel formulation, which is not prepared to reproduce the material work softening. Therefore, our findings provide evidence that the flow stress dependence on temperature is well-established in the incremental model, which accurately reproduces the wall thickness eccentricity imperfection. As a result, it can be concluded that both models are able to reproduce the RPT process, but the incremental model reproduces the softening of the material and provides extra information regarding the active restoration mechanisms, aimed at achieving a complete understanding of the material deformation during the RTP process.

**Figure 3:**
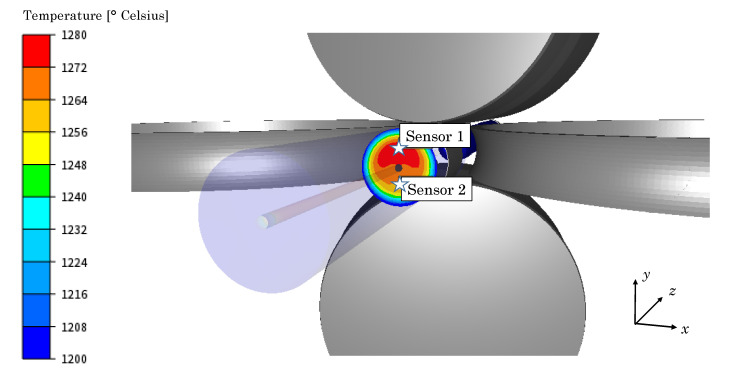
Position of the sensors 1 and 2 in the cross-section of the billet.

Hereafter, the incremental formulation is used to investigate the work softening of the material during the RTP process. This type of analysis cannot be accomplished with most commonly used phenomenological material models, due to its inherent formulation. In order to discern which deformation mechanisms are involved, the deformation path experienced by the material during the RTP process is analyzed. To this end, two Lagrangian sensors were placed in the middle length of the radius of the cross-section of the billet, in a similar diameter, but in opposite directions as illustrated in Figure 3.

**Figure 4:**
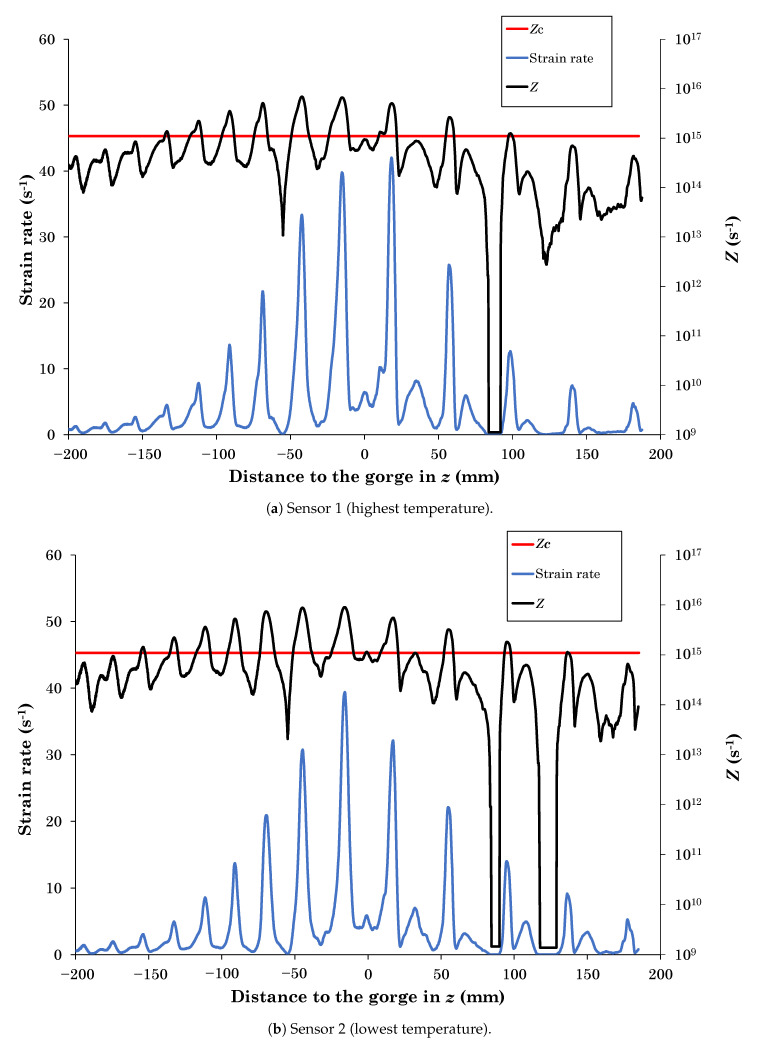
Evolution of the Zener–Hollomon parameter *Z* and the strain rate with respect to the distance to the gorge.

Sensor 1 was set in the part of the cross-section with higher temperature while Sensor 2 was set in the region with lower temperature. As previously stated in the Section 2, the Zener–Hollomon parameter can be used to determine if the material undergoes DRV $\left(Z\geq Z_{c}\right)$ or if DRX is the prevailing restoration mechanism $\left(Z<Z_{c}\right)$. In Figure 4, the evolution of the Zener–Hollomon parameter and material strain rate in both sensors with respect to the point where the rolls are at their minimum distance (gorge) is given.

The evolution of the Zener–Hollomon parameter in both sensors (black curves) is similar with respect to the critical value $Z_{c}$ (red line). Sensor 1 was placed at a higher temperature than Sensor 2, but also presents higher strain rate (blue curves). Therefore, the effect of temperature and strain rate on *Z* is compensated and thus both sensors show similar values of *Z*. As long as *Z* overcomes the critical value $Z_{c}$, the DRV is the only active restoration mechanism, while at $Z<Z_{c}$, DRX is the prevailing restoration mechanism. Therefore, Figure 4a,b confirm how both restoration mechanisms are continuously fluctuating while the billet is advancing in the piercing direction *z*.

**Figure 5:**
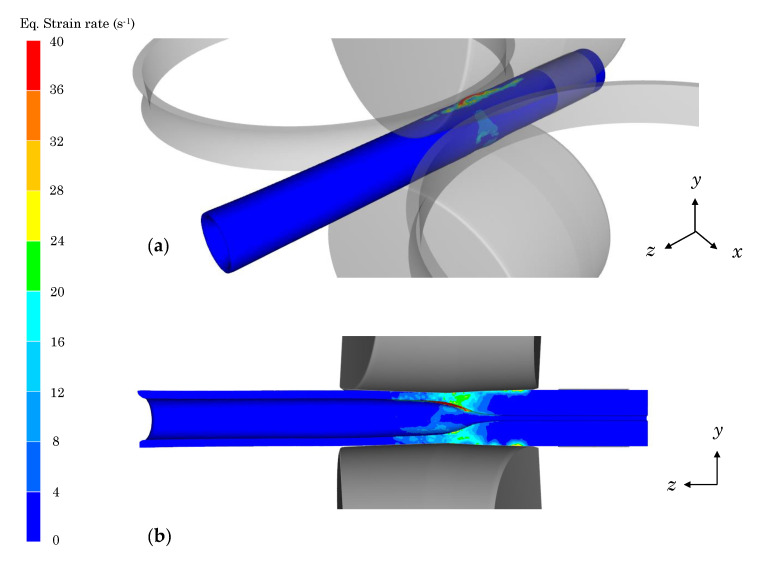
Strain rate distribution during the rotary tube piercing (RTP) process: (**a**) isometric projection, (**b**) *z–y* plane.

The strain rate of the material during the RTP process is given in Figure 5.

The peaks in the strain rates of Figure 4a,b correspond to the instant when the material is in contact with the rolls (see Figure 5). At this time, the material is accelerated, which leads to a sudden increase in its deformation, reaching strain rates up to 40 s^−1^. *Z* is dependent on the strain rate of the material and thus, in this moment $Z\geq Z_{c}$, DRV occurs, and the material does not soften due to DRX. However, when the material rotates between the upper and lower rolls, it only contacts the Dieschers that restrict the rotation. At this period, the strain rate values are low and thus, DRX is taking place, which softens the material. Finally, when the hollow cavity of the tube has been developed and the material does not contact the Dieschers (z≥75 mm), the strain rate tends to be null at certain points where there is no contact with the rolls (see Figure 4a,b). At this period, *Z* presents values under $Z_{c}$ but it is not dynamically restoring because $\dot{\bar{\epsilon }}\approx 0$.

Regarding the temperature evolution, the plastic deformation energy of the material is converted into heat, which leads to an average increase of the material temperature of approximately 50 °C during the RTP process. This increase of temperature yields the decrease of *Z*. It can be appreciated in Figure 4a,b at the positions z=−100 mm and z=100 mm. In both cases, the material contacts the roll and there is a peak in the strain rate value of similar magnitude. However, as in the position z=100 mm, where the material was heated, the magnitude of *Z* was lower than at the position z=−100 mm. Nevertheless, the heating of the material does not show a strong effect on the determination of which restoration mechanism is acting as it does the strain rate.

**Figure 6:**
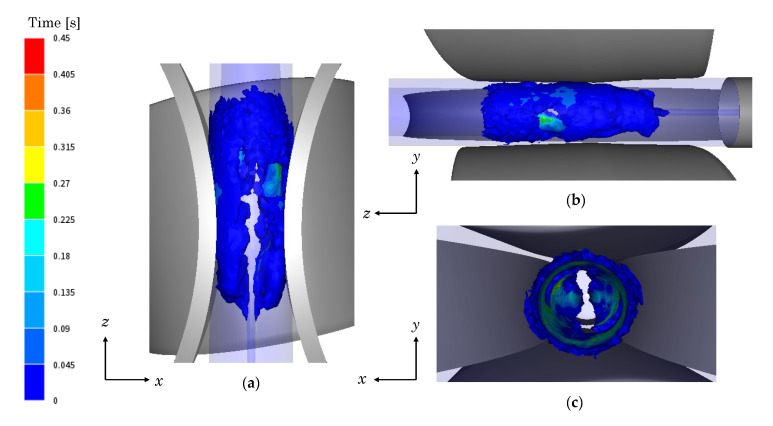
Time during which the material exhibits dynamic recrystallization (DRX) in the RTP process: (**a**) upper view, (**b**) lateral view and (**c**) front view.

Finally, the location of the material volume that is undergoing DRX is assessed. In Figure 6, the region of the material that underges DRX is highlighted in the tube during the RTP process. The colours represent the time when the material is restored by DRX uninterruptedly.

It can be seen that in the periphery of the tube, blue color prevails, which indicates low duration of uninterrupted DRX restoration. This is in agreement with the above-explained, regarding the fluctuation of DRX and DRV mechanisms due to the high strain rate occurring at the contact of the tube with the rolls. In contrast, in the inner part of the tube, the DRX restoration times are higher since this region is less affected by the effect of the rolls.

## 5. Conclusions

In this paper, an incremental model of the P91 steel has been implemented in a commercial FE software to describe the flow stress of the material in the RTP process. The adequacy of the model for such complex process has been assessed and the material dynamic restoration mechanisms, namely, DRV and DRX, have been analyzed. The following conclusions have been drawn from this study:

- The application of the constitutive model on the simulation of the RTP process leads to the accurate reproduction of the tube geometry parameters, namely, elongation, longitudinal torsion, average wall thickness and wall thickness eccentricity.
- The constitutive model dependence on the temperature is well-established at the sight of the precise wall thickness eccentricity results obtained.
- In contrast to the previously used formulations, the proposed model allows analyzing the material restoration mechanisms involved in the RTP process. The DRV and DRX are found to prevail over each other cyclically, following an alternating sequence, during the material processing.
- The material strain rate is the most dominant parameter in the determination of the prevailing dynamic restoration mechanism.
- DRV prevails when the material is in contact with both rolls and plug, otherwise DRX is the main restoration mechanism.
- The influence of material DRX on the wall thickness eccentricity is limited, because the eccentricity is produced in the roll–material and plug–material contacts. As previously reported, the material DRV prevails at this region.

## Figures and Tables

**Table 1:** Material parameter values [56].

$\sigma_{a}$	$\delta_{Y}$	$B_{Y}$	$m_{Y}$	$k_{\theta }$	$m_{\theta }$	$\delta_{S}$
3.7	28	$3.52\times 10^{13}$	4.5	$9\times 10^{-4}$	$7.5\times 10^{-2}$	116.9
$B_{S}$	$m_{S}$	$\delta_{\mathrm{SS}}$	$B_{\mathrm{SS}}$	$m_{\mathrm{SS}}$	*D*	*q*
$5.2\times 10^{15}$	5.5	1260	$3.34\times 10^{20}$	4.5	0.5	0.97

**Table 2:** Parameters of the rotary piercing mill FE model [29].

Parameters	Magnitude
Cross angle, δ	3°
Feed angle, β	10°
Roll diameter	900 mm
Roll angular velocity	111 rpm
Diescher diameter	1700 mm
Diescher angular velocity	24 rpm

**Table 3:** Comparison of deformation numerical and experimental results using the incremental and Hansel–Spittel constitutive models.

Result	Experiment	Incremental Model		Hansel–Spittel	
		Result	Difference (%)	Result	Difference (%)
$\epsilon_{z}$	2.08	2.10	0.96	2.13	2.4
dψ/dz	52.1 °C/m	44 °C/m	15.6	54.8 °C/m	5.18
$t_{tubeavg}$	27.75 mm	27.83 mm	0.29	27.41 mm	1.23
$e_{c}$	3.75%	3.84%	2.40	5.44%	45.1
